# The enhanced expression of the matrix metalloproteinase 9 in nasal NK/T-cell lymphoma

**DOI:** 10.1186/1471-2407-7-229

**Published:** 2007-12-19

**Authors:** Koh-ichi Sakata, Masanori Someya, Mutsuko Omatsu, Hiroko Asanuma, Tadashi Hasegawa, Masato Hareyama, Tetsuo Himi

**Affiliations:** 1 Department of Radiology, Sapporo Medical University, School of Medicine, Sapporo, Japan; 2 Department of Surgical Pathology, Sapporo Medical University, School of Medicine, Sapporo, Japan; 3 Department of Otorhinolaryngology, Sapporo Medical University, School of Medicine, Sapporo, Japan

## Abstract

**Background:**

Nasal NK/T cell lymphoma is an aggressive disease and has a poor prognosis. Nasal NK/T cell lymphoma is refractory to conventional chemotherapy and has strong tendency of widespread relapse or dissemination into distant sites.

**Methods:**

We immunohistochemically studied nasal NK/T-cell lymphoma to elucidate the unique characteristics of nasal NK/T-cell lymphoma, such as its higher metastatic tendency and its vast necrosis which leads to destruction of the involved tissues. The expression of P-glycoprotein and MMP-9 was evaluated in the 20 patients with nasal NK/T-cell lymphoma and 25 with nasal non-NK/T-cell lymphoma and the relationship between expression of these proteins and clinical results were analyzed in this report.

**Results:**

Overall 5-year survival rates for patients with nasal NK/T cell lymphoma, and nasal non-NK/T cell lymphoma were 51%, and 84%. Distant involvement free 5-year survival rates for patients with nasal NK/T cell lymphoma, and nasal non-NK/T cell lymphoma were 53%, and 79%.

Overall positivity for P-glycoprotein was observed in 10 of 19 patients with NTL and in 13 of 23 patients with non-NTL. When the overall survival rate was compared between patients with P-glycoprotein positive and negative, there was no difference between them.

Sixteen of the 19 patients with nasal NK/T cell lymphoma expressed MMP-9. In contrast, only 8 of the 22 patients with nasal non-NK/T cell lymphoma expressed MMP-9. Distant involvement free 5-year survival rates for patients with MMP-9 negative, and MMP-9 positive were 92%, and 61%, respectively. The difference was statistically significant (p = 0.027).

**Conclusion:**

Positive immunoreactivity for P-glycoprotein was not an independent prognostic factor in nasal NK/T-cell lymphomas, which stresses the importance of exploring other mechanisms of drug resistance. The strong expression of MMP-9 is uniquely characteristic of nasal NK/T cell lymphoma and may contribute to its strong tendency to disseminatate and the extensive necrosis which is always seen. However, our results are based on univariate comparisons, and as such, should be viewed with some caution.

## Background

Extranodal NK/T-cell lymphoma, nasal type (nasal NK/T cell lymphoma) is characterized by progressive, unrelenting ulceration, and necrosis of the nasal cavity and midline facial tissues. This subtype of lymphoma, categorized as angiocentric lymphoma in the Revised European-American Lymphoma (REAL) classification [1], has been renamed extranodal NK/T-cell lymphoma, nasal type in the new WHO classification of lymphoid neoplasms [2]. This type of lymphoma is rare in the United States and Europe but common in Asia [3-6]. The prognosis of Asian population as well as Central American population is not so poor as European population [7]. Phenotypically, this lymphoma is characterized by expression of NK cell marker CD56, lack of expression of surface CD3, infrequent expression of T-lineage markers other than CD2, absence of T-cell receptor rearrangement, and strong association with Epstein-Barr virus (EBV) [6,8].

Nasal NK/T cell lymphoma is an aggressive disease and has a poor prognosis. One of reasons for the poor prognosis of nasal NK/T cell lymphoma is that NK/T-cell lymphomas are refractory to conventional chemotherapy [9-11]. Doxorubicin-containing chemotherapy regimens generally appear less efficacious, either as an initial treatment or as a salvage treatment for relapsed lesions in the management of NK/T-cell lymphoma patients [10]. The ineffectiveness of chemotherapy for such lymphoma types can be partly explained by the presence of the multi-drug resistance (MDR) phenotype, which confers cellular resistance to a variety of unrelated anticancer agents [10-12]. Despite accumulating experimental evidence that high levels of P-glycoproteins are commonly expressed in malignant tumor cells of various types of NK-neoplasms [12,13], convincing data is very limited, largely due to the rarity of these types of lymphomas [14].

Another reason for the poor prognosis of nasal NK/T cell lymphoma is its strong tendency of widespread relapse or dissemination into distant sites. Essential steps in the process of tumor invasion and metastasis include the degradation of the extracellular matrix (ECM) and basement membrane (BM). The invasion of the BM by tumor cells is thought to be one of the critical steps in metastasis, which includes sequential multistep processes [15]. Many proteolytic enzymes degrade components of the ECM and BM [16,17]. Among these, the matrix metalloproteinases (MMPs) are attractive candidates as enzymes required for tumor metastasis. The MMPs contain a zinc ion at their active sites and can degrade native collagens and other ECM components [18,19]. The MMP family includes four types of collagenase (MMP-1, -8, -13, and -18), three types of stromelysin (MMP-3, -10, and -11), and the 72- and 92-kDa type IV gelatinases or collagenases (MMP-2 and MMP-9). As type IV collagen is one of the integral components of BM, the uncontrolled expression of two type IV collagenases, MMP-9, is believed to play a critical role in the invasion of BM by tumor cells [20]. The release of MMP-9 has been associated with metastasis in a variety of model systems [21-24]. MMP-9 can be also responsible for chemoresistance of NK/T-cell lymphoma. Higher expression of MMP-9 could result in tissue necrosis due to more angiodestruction. Poor drug delivery owing to tissue necrosis might be an important contributory factor [8].

In the current study, we performed an immunohistochemical study of the expression of P-glycoprotein and MMP-9 in nasal NK/T-cell lymphoma and other types of nasal lymphomas (non-NK/T cell lymphoma) to elucidate the unique characteristics of nasal NK/T-cell lymphoma, such as its resistance of chemotherapy and strong metastatic tendency.

## Methods

### Population

A total of 45 patients with nasal lymphoma were treated in our institution between January 1980 and December 2005. The patient population consisted of 20 patients (10 men, 10 women) with nasal NK/T cell lymphoma, and 25 patients (12 men, 13 women) with nasal non-NK/T cell lymphoma (Table 1). Cases with nasal non-NK/T cell lymphoma consisted of 18 patients with diffuse large B-cell lymphoma, one with B-cell small lymphocytic lymphoma, 3 with peripheral T-cell lymphoma, unspecified, one with follicular lymphoma, adult T-cell Lymphoma, and anaplastic large cell, T-cell lymphoma according to the WHO classification system. Patients were usually followed every 2 months for the first 2 years and every 3 to 5 months subsequently. Physical and endoscopical examination were performed at every visit and CT were usually performed every 6 months. The median follow-up period of surviving patients was 94 months.

**Table 1:** Characteristics of patients

	NTL	non-NTL
Patient number	20	25
Median age (range)	56 (27–74)	59 (21–81)
Sex (male:female)	10:10	12:13
Stage		
I	14	14
II	4	6
III, IV	2	4
B symptoms	14	2
Yes	14	2
No	6	23
EBER		
Yes	18	6
No	2	19

### Treatment of the primary tumor

The radiation portal encompassed only clinically involved areas with a generous margin in most patients and prophylactic irradiation to the neck and supraclavicular lymph nodes was not usually performed. The median dose received was 40 Gy (range 9–74 Gy). The radiation doses were heterogeneous because the radiation was interrupted in 2 patients with nasal NK/T cell lymphoma due to deterioration of general condition and becuase in 1990 the standard radiation dose was increased from 40 Gy to 50 Gy for patients with nasal NK/T cell lymphoma.

From 1980 to 1986, radiation therapy alone was the primary treatment, although adjuvant chemotherapy was given to some patients. Since 1987, a combination of radiotherapy and chemotherapy has been the standard treatment. The main chemotherapy combinations before 1985 did not include adriamycin, e.g. COP (cyclophosphamide, vincristine, and prednisolone) or VEMP (vincristine, cyclophosphamide, methotrexate and prednisolone). These were administered after radiotherapy. Most patients from 1985 on received CHOP (cyclophosphamide, doxorubicin, vincristine, and prednisolone), VEPA (same drugs as CHOP, but doses and treatment schedule were different), or MACOP-P (methotrexate, doxorubicin, cyclophosphamide, vincristine, prednisolone, and pepleomycin) had been used before and after radiotherapy since 1989.

### Statistical Analysis

All but 3 surviving patients had a minimum follow-up of 2 years and the follow-up periods of the other 3 patients were, 1, 1, and 4 months. The median follow-up of surviving patients was 94 months. Survival rates of the patients were measured using the Kaplan-Meier method. The overall survival was calculated from the date when the treatment started to the time of death or last follow-up. The distant involvement free survival was calculated from the date when the treatment started to the time of diagnosis of distant metastasis or last follow-up. Statistical significance was compared by the log rank test.

### Immunohistochemical Examination

All of the biopsies were taken at the initial time of the diagnosis. Immunohistochemical staining was carried out with methods previously described [25]. All of the biopsies were taken at the initial time of the diagnosis. We used biopsy samples of 43 patients and samples of 3 patients were not evaluable because they were too tiny. Immunohistochemical staining was carried out using the avidin-biotin-peroxidase complex method.

Immunohistochemical detection was performed using the following monoclonal antibodies: antihuman MMP-9 (56-2A4, Daiichi Fine Chemical Co., Ltd., Japan)(1: 200 dilution), and MDR/P-glycoprotein antibody (C-494, Dako; 1:100 dilution). Normal mouse serum was substituted for primary antibodies as a negative control.

The specificity and selectivity of antibody of MMP-9 was proved by Fujimoto et al. and this manufacturer [26]. For MMP-9, tumors that contained at least focally moderate to strong immunoreactivity were considered positive.

To detect P-glycoprotein, we selected mouse anti-human C-494 antibody, which recognizes an internal epitope located on the C-terminal domain of the P-glycoprotein molecule, and which is encoded by two closely related genes, MDR1 and MDR3. Tumors were considered positive for P-glycoprotein expression if the neoplastic cells exhibited membrane staining greater than 10%, according to a cutoff value that has been recommended in the majority of immunohistochemical studies for B-cell lymphomas [27].

### In situ hybridization

RNA-DNA in situ hybridization was performed on formalin-fixed paraffin-embedded tissue sections using the biotinylated synthetic DNA probe as previously described with modifications [28]. Briefly, the biotinylated probe was an oligonucleotide DNA complimentary to EBER1 sequence that was chemically labeled with 6 biotin molecules (5'-CCCTAGCAAAACCTCTAGGGCAGC-(TAG)5-BBB-(TAG)2-BBB-3'). The hybridization signal was detected by using ABC-peroxidase. The sections were then counterstained with hematoxylin, dehydrated, mounted with permount, and investigated under a light microscope.

## Results

**Figure 1:**
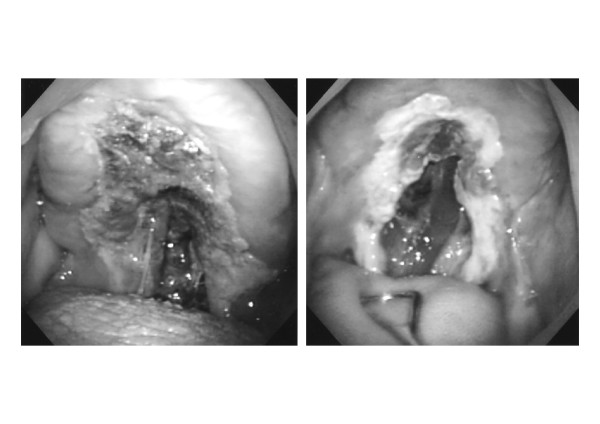
There was a deep ulcer in the middle of the hard palate with foul-smelling discharge (left photo). After radiotherapy, the tumor disappeared and deficit of the anterior palate appeared (right photo).

Clinically, nasal NK/T cell lymphoma had unique features characterized by destruction of the nasal cavity and midline facial tissues caused by progressive and unrelenting ulceration and necrosis of the involved tissues, as demonstrated in Fig. 1. This destructive feature of nasal NK/T cell lymphoma is very characteristic, and distinct from the other types of malignant lymphoma, which form tumors or cause swelling of the involved lymph nodes. A characteristic feature is invasion of vascular walls and, usually occlusion of lumina by lymphoid cells. The vascular occlusion is usually associated with prominent ischemic necrosis of both tumor cells and normal tissue.

Most patients (70%) with nasal NK/T cell lymphoma had B symptoms, such as fever or unexplained loss of 10% or more of their body weight in the six months before diagnosis. In contrast, only less than10% of patients with nasal non-NK/T cell lymphoma had B symptoms (Table 1). EBER1 transcripts were detected in 18 of 20 patients with nasal NK/T cell lymphoma (Table 1), indicating strong association with Epstein-Barr virus (EBV).

**Figure 2:**
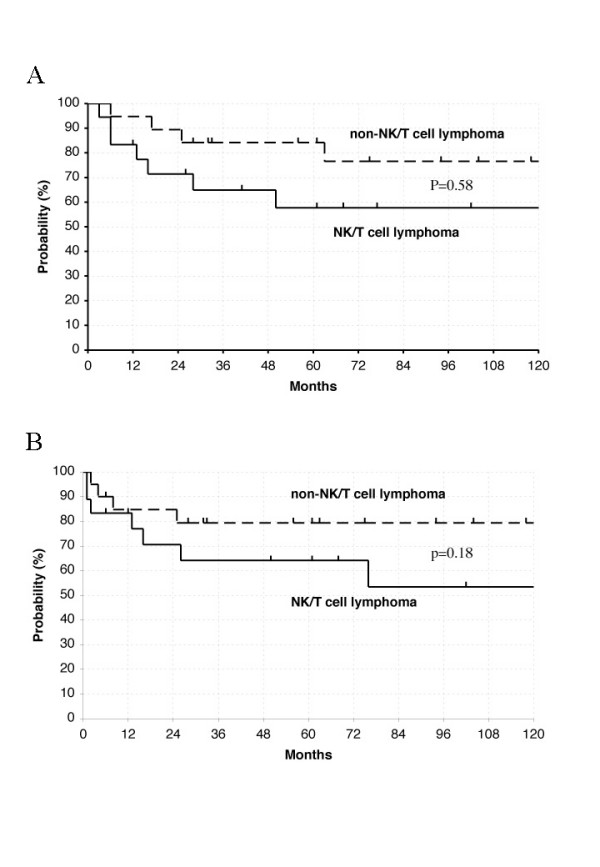
(a) The overall survival rates of patients of stage I or II with nasal NK/T cell lymphoma and nasal non-NK/T cell lymphoma. (b) The distant involvement free rates of patients of stage I or II with nasal NK/T cell lymphoma and nasal non-NK/T cell lymphoma.

Overall 5-year survival rates for patients with nasal NK/T cell lymphoma, and nasal non-NK/T cell lymphoma were 51%, and 84% (p = 0.58), respectively (Fig. 2a). The clinical courses were quite aggressive and nasal NK/T cell lymphoma patients have a higher incidence of widespread extranodal involvement, even though their stage was only I or II. Distant involvementfree 5-year survival rates for patients with nasal NK/T cell lymphoma, and nasal non-NK/T cell lymphoma were 53%, and 79% (p = 0.18), respectively (Fig. 2b). The difference is not statistically significant, however, this is most likely because of the small sample size.

**Figure 3:**
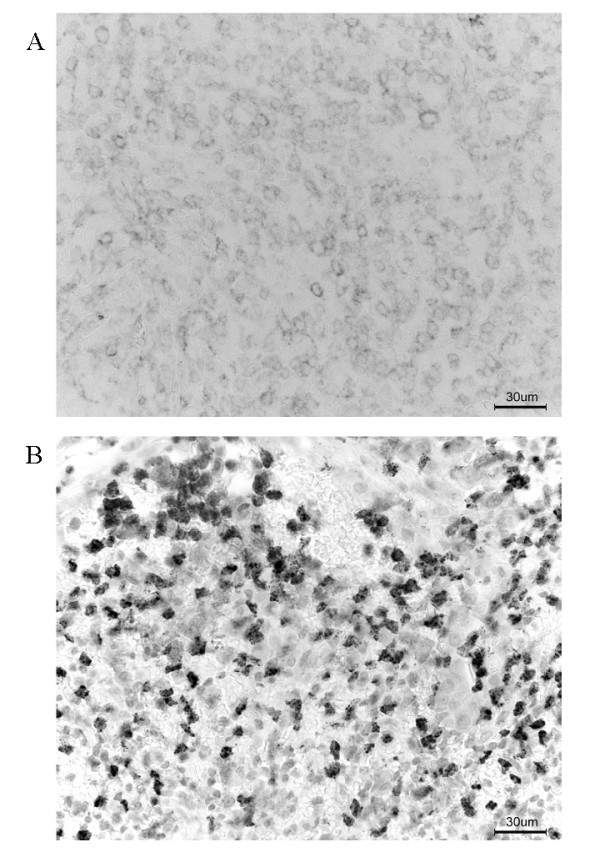
(a) Expression of P-glycoprotein in nasal NK/T cell lymphoma (original magnification, ×200). (b) Expression of MMP-9 in nasal NK/T cell lymphoma (original magnification, ×200).

**Figure 4:**
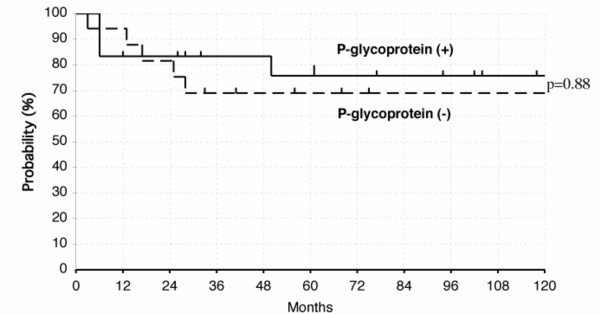
The overall survival rates of patients with B and T-cell lymphomas of stage I or II with P-glycoprotein positive or negative. All classifications of B and T-cell lymphomas were joined together and analyzed.

Within malignant cells, patterns of P-glycoprotein immunoreactivity generally were of membrane staining, but cytoplasmic immunoreactivity was occasionally observed in some tumor cells (Fig. 3a). When a staining distribution cutoff value of greater than 10% was used to classify positive immunoreactivity, overall positivity for P-glycoprotein was observed in 10 of 19 patients with NTL and in 13 of 23 patients with non-NTL (Table 2). When the overall survival rate was compared between patients with P-glycoprotein positive and negative, there was no difference between them (p = 0.88) (Fig. 4). The similar result was obtained, when this analysis was also done separately for T and B cell lymphomas. The 5-year survival rate for P-glycoprotein positive and negative in T cell lymphomas was 68% and 67%. The 5-year survival rate for P-glycoprotein positive and negative in B cell lymphomas was 85% and 71%.

**Table 2:** Expression of P-glycoprotein according to WHO classification

	positive	negative
NTL	10	9
Peripheral T cell lymphoma, unspecified	2	1
Anaplastic large cell, T-cell lymphoma	0	1
Adult T-cell Lymphoma	1	0
DLBCL	8	8
Follicular lymphoma	1	0
B-cell small lymphocytic lymphoma	1	0

**Figure 5:**
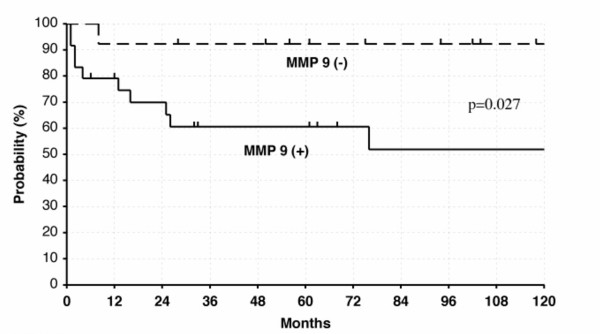
The distant involvement free rates of patients with B and T-cell lymphomasof stage I or II with MMP9 positive or negative. All classifications of B and T-cell lymphomas were joined together and analyzed.

To identify the localization of the MMP-9 gene product, immunohistochemical staining was performed on biopsy sections of patients with NK/T cell lymphoma and non-NK/T cell lymphoma (Fig. 3b, Table 3). Sixteen of the 19 patients with nasal NK/T cell lymphoma expressed MMP-9. In contrast, only 8 of the 22 patients with nasal non-NK/T cell lymphoma expressed MMP-9. Immunohistochemically, the MMP-9 gene product was localized in lymphoma cells, macrophages, and neutrophils. All cells that were positive for MMP-9 were intensely stained, indicating that they expressed high levels of MMP-9. Cells which expressed MMP-9 existed at the invasive edge of tumor cell nests and the peripheral regions of the necrotic zone in nasal NK/T cell lymphomas. Distant involvement free 5-year survival rates were compared between patients with MMP-9 positive and negative (Fig. 5). Distant involvement free 5-year survival rates for patients with MMP-9 negative, and MMP-9 positive were 92%, and 61%, respectively. The difference was statistically significant (p = 0.027). The similar result was obtained, when this analysis was also done separately for T and B cell lymphomas. Distant involvement free 5-year survival rates for patients with MMP-9 negative, and MMP-9 positive in T cell lymphomas were 89%, and 57%, respectively. Distant involvement free 5-year survival rates for patients with MMP-9 negative, and MMP-9 positive in B cell lymphomas were 100%, and 62%, respectively.

**Table 3:** Expression of MMP9 according to WHO classification

	positive	negative
NTL	16	3
Peripheral T cell lymphoma, unspecified	2	1
Anaplastic large cell, T-cell lymphoma	0	1
Adult T-cell Lymphoma	0	1
DLBCL	5	11
Follicular lymphoma	0	1
B-cell small lymphocytic lymphoma	1	0

## Discussion

The overall outcome of nasal NK/T cell lymphoma is poor, even if diagnosed in the early stage [9,29,30]. One of the reasons for the poor prognosis of nasal NK/T cell lymphoma is its resistance to chemotherapy. Cheung et al. reported that of 61 patients who were administered chemotherapy, 31 showed disease progression while receiving chemotherapy, of whom 17 progressed locoregionally. They concluded that the addition of anthracycline-containing chemotherapy to radiotherapy does not appear to confer any survival benefit in stage I patients [8]. The reasons for the resistance to chemotherapy are not clear. The overexpression of MDR1 phenotype is considered one of the major determinants for the ineffectiveness of chemotherapy in nasal NK/T-cell lymphoma patients [10,11]. However, there was no significant difference in overall positivity for P-glycoprotein between NTL and non-NTL in our study (Table 2). Positive immunoreactivity for P-glycoprotein was not found to be an important prognostic factor for patients' survival (Fig. 4). Kim et al. reported similar results obtained by using the same antibody, mouse anti-human C-494 antibody on paraffin-embedded sections [14]. These results indicate that the frequent expression of MDR (P-glycoprotein-positive) phenotype may account for a certain proportion [12], but not all, of the failure of chemotherapy [31]. Necrosis is also a constant microscopical feature in nasal NK/T cell lymphoma, usually with a zonal pattern of distribution that suggests a vascular pathogenesis. Poor drug delivery owing to tissue necrosis resulting from angiodestruction by nasal NK/T cell lymphoma cells might be an important contributory factor [8].

Another reason for the poor prognosis of nasal NK/T cell lymphoma is its strong tendency to widespread relapse or to disseminate into distant sites. Common sites of distant involvement during progression of the disease were systemic lymph nodes, lung, liver, spleen, skin, and gastrointestinal tracts [32]. We found that most patients with nasal NK/T cell lymphoma expressed high levels of MMP-9. Release of MMP-9 enables tumor cells to invade through basement membranes of blood vessels and lymphatics, thereby initiating metastatic spread [16,33]. Thus, the strong expression of MMP-9 in nasal NK/T cell lymphoma may explain its strong tendency to metastasize.

We also reported the expression of MMP9 in 158 patients with non-Hodgkin's lymphomas [34]. Almost all of the patients with nasal NK/T cell lymphoma and anaplastic large-cell lymphoma expressed MMP9. In this study, 15 of 17 (88%) patients of nasal NK/T cell lymphoma expressed MMP9. Five of 8 (63%) of peripheral T-cell lymphomas, unspecified expressed MMP9 and 4 of 4 (100%) of peripheral T-cell lymphomas, unspecified expressed MMP9. In contrast, only a small fraction of the patients with exranodal marginal zone B-cell lymphoma of mucosa-associated lymphoid tissue type and follicular lymphomas expressed MMP9. About 50% of the diffuse large B-cell lymphomas (DLBCL) expressed MMP9. Overall survival rates of patients who expressed MMP9 were significantly lower than those who did not.

Inhibition of the function of MMPs is being pursued for anticancer therapy. TIMPs (tissue inhibitors of metalloprotease) were first compounds to be considered for clinical development. However, the lack of effective methods of systemic gene delivery has limited the clinical utility of this approach, whereas the development of synthetic inhibitors of MMPs has been actively pursued and widely tested in clinical trials [35].

Progress in the treatment for nasal NK/T lymphomas has been slow due to the rarity of the diseases, geographic variation, relative chemoresistance, and lack of randomized trials. There is no consensus about optimal therapy and recommendations are based on anecdotal reports, small series, and phase II trials. There is general agreement that results with CHOP alone are so poor in adults with most peripheral T/NK cell lymphomas [36]. So, the combined radiotherapy and chemotherapy was used [37]. We previously reported 65 patients with mature T/NK-cell lymphomas treated with radiotherapy between 1983 and 2002 to analyze the influence of radiotherapy doses and chemotherapy doses and clinical parameters on in-field disease control in order to assess the optimal radiation doses for treatment of mature T/NK-cell lymphomas [38]. There were no significant differences in radiosensitivity among subtypes of mature T/NK-cell lymphomas, at least between nasal NK/T cell lymphoma and peripheral T-cell lymphomas, unspecified. Radiation doses of 50 Gy or more may be required to obtain in-field control of mature T/NK-cell lymphomas [38].

## Conclusion

In conclusion, positive immunoreactivity for P-glycoprotein was not an independent prognostic factor in nasal NK/T-cell lymphomas. Although the role of strong MMP-9 expression in the spread of nasal NK/T cell lymphoma has yet to be definitely established, our findings demonstrated that MMP-9 may be related to a strong tendency to metastasize and the locally destructive nature of nasal NK/T cell lymphoma. However, our results are based on univariate comparisons, and as such, should be viewed with some caution.

## Competing interests

The author(s) declare that they have no competing interests.

## Authors' contributions

KS and MS carried out immunohistochemical examinations and drafted    the manuscript. MH and TH (Tetsuo HiMi) treated patients and    statistical analyses. MO, HA, and TH (Takashi Hasegawa) performed the    histological diagnosis and in situ hybridization. All authors read    and approved the final abstract.  

## Pre-publication history

The pre-publication history for this paper can be accessed here:


