# Correction: The enhanced expression of the matrix metalloproteinase 9 in nasal NK/T-cell lymphoma

**DOI:** 10.1186/1471-2407-8-45

**Published:** 2008-02-07

**Authors:** Koh-ichi Sakata, Masanori Someya, Mutsuko Omatsu, Hiroko Asanuma, Tadashi Hasegawa, Shingo Ichimiya, Masato Hareyama, Tetsuo Himi

**Affiliations:** 1 Department of Radiology, Sapporo Medical University, School of Medicine, Sapporo, Japan; 2 Department of Surgical Pathology, Sapporo Medical University, School of Medicine, Sapporo, Japan; 3 First Department of Pathology, Sapporo Medical University, School of Medicine, Sapporo, Japan; 4 Department of Otorhinolaryngology, Sapporo Medical University, School of Medicine, Sapporo, Japan

## 

Following the publication of the article 'The enhanced expression of the matrix metalloproteinase 9 in nasal NK/T-cell lymphoma. BMC Cancer 2007, 7:229' [1], the submitting author became aware that a co-author had been omitted from the publication. Therefore, this article has been submitted as a correction to the original text, and highlights the contributions that this author made to the original article. The author would like to apologize for any inconvenience this may have caused.

## Corrected author list

Koh-ichi Sakata, Masanori Someya, Mutsuko Omatsu, Hiroko Asanuma, Tadashi Hasegawa, Shingo Ichimiya, Masato Hareyama, and Tetsuo Himi.

## Corrected authors' contributions

KS and MS carried out immunohistochemical examinations and drafted the manuscript. MH and TH (Tetsuo HiMi) treated patients and statistical analyses. MO, HA, and TH (Takashi Hasegawa) performed the histological diagnosis and in situ hybridization. SI improved the method to detect EBER in the nucleus of nasal T cell lymphoma cells by RNA-DNA in situ hybridization and attended pathological diagnosis of nasal T cell lymphoma. All authors read and approved the final abstract.

## Pre-publication history

The pre-publication history for this paper can be accessed here:


